# A parametric method for cumulative incidence modeling with a new four-parameter log-logistic distribution

**DOI:** 10.1186/1742-4682-8-43

**Published:** 2011-11-11

**Authors:** Zahra Shayan, Seyyed Mohammad Taghi Ayatollahi, Najaf Zare

**Affiliations:** 1 Department of Biostatistics, Shiraz University of Medical Sciences, Shiraz, Iran

## Abstract

**Background:**

Competing risks, which are particularly encountered in medical studies, are an important topic of concern, and appropriate analyses must be used for these data. One feature of competing risks is the cumulative incidence function, which is modeled in most studies using non- or semi-parametric methods. However, parametric models are required in some cases to ensure maximum efficiency, and to fit various shapes of hazard function.

**Methods:**

We have used the stable distributions family of Hougaard to propose a new four-parameter distribution by extending a two-parameter log-logistic distribution, and carried out a simulation study to compare the cumulative incidence estimated with this distribution with the estimates obtained using a non-parametric method. To test our approach in a practical application, the model was applied to a set of real data on fertility history.

**Conclusions:**

The results of simulation studies showed that the estimated cumulative incidence function was more accurate than non-parametric estimates in some settings. Analyses of real data indicated that the proposed distribution showed a much better fit to the data than the other distributions tested. Therefore, the new distribution is recommended for practical applications to parameterize the cumulative incidence function in competing risk settings.

## Background

In medical research with time-to-event data, there may be more than one final outcome of interest, and this circumstance can complicate the statistical analysis. In such cases, events other than the desired one(s) are considered as competing risks when their occurrence prevents the event of interest [1,2]. An important quantity in competing risk settings is the cumulative incidence function (CIF), which makes it possible to calculate the probability of a particular event. In contrast, the cause-specific hazard function (CSHF) calculates the instantaneous rate of the event. For example, in fertility studies in women, researchers are interested in calculating the cumulative live birth rate in the presence of competing risks over time. Competing events, such as the probability of stillborn fetuses or abortions, can be calculated.

Most competing risk analyses of CIF are estimated non- or semi-parametrically [3,4]. However, the parametric model is another available approach for modeling CIF. The advantage of parametric methods compared to non- and semi-parametric ones is that if a parametric model is selected correctly, it can predict the probability of the occurrence of events in the long term and provide additional insights about the time to failure and hazard functions [5]. Also, when the survival pattern follows a particular parametric model, the estimates from true model fit are usually more accurate than the non-parametric estimates.

The best known distributions for modeling CIF are the Weibull and Gompertz distributions. However, these are suitable only for hazard functions that increase or decrease monotonically; they are inadequate when the hazard function shape is unimodal. In such cases, simple distributions such as the two-parameter log-logistic or log-normal distributions are likely to be better choices. One approach to the construction of flexible parametric models is to add a shape parameter to provide a wide range of hazard shapes and improve the models in survival data. In 1996, Mudholkar *et al*. proposed a generalized Weibull family with a range of hazard shapes [6] and Foucher *et al*. in 2005 applied this distribution in semi-Markov models [7]. In 2006, Sparling *et al*. presented a three-parameter family of survival distributions that included the Weibull, negative binomial, and log-logistic distributions as special cases [8]. These distributions can fit U-shapes or unimodal shapes for the hazard function, and therefore can be appropriate for survival data.

In light of the issues summarized above, a more efficient parametric distribution with various shapes of hazard patterns would appear to be useful for estimating CIF in competing risk situations. In recent years, various parametric distributions have been developed specifically for analyzing competing risk data that offer more flexibility. For example, in 2006 Jeong introduced a new parametric distribution for modeling CIF [5]. In 2009, Wahed *et al*. developed Weibull's distribution, resulting in a beta-Weibull four-parameter distribution for use in competing risks [9]. Here, we propose a new four-parameter log-logistic distribution by extension of a two-parameter log-logistic distribution that contains different kinds of hazard shapes in survival data and increases the efficiency of the CIF over the non-parametric approaches. Also, this is an improper distribution which enjoys more flexibility for modeling of CIF. Therefore, it would be suitable for competing risk models. We have performed a simulation study to compare CIF estimates obtained with the four-parameter distribution and a non-parametric method. After using simulated data to assess the method, we analyzed a real data set to examine the efficiency of our proposed distribution.

## Methods

### Introduction of the new distribution

The survival function according to a two-parameter log-logistic distribution is as follows:

(1) $$S\left(t\right)=\frac{1}{1+\lambda t^{\tau }}$$

where *λ *> 0 and *τ *> 0 are the scale and shape parameters, respectively. If *τ *≤ 1, the hazard function decreases monotonically, whereas if *τ *> 1, the hazard function is unimodal [10].

### Survival function of the four-parameter log-logistic distribution

The two-parameter log-logistic distribution is expanded on the basis of the family of Hougaard stable distributions, whose survival function is as follows:

(2) $$\begin{array}{c} S\left(t\right)=\\ \exp\left\{-\frac{\upsilon \theta^{\alpha }}{\alpha }\left[{\left(\frac{H}{\theta }+1\right)}^{\alpha }-1\right]\right\} \end{array}$$

where H is the cumulative hazard function [11]. If a two-parameter log-logistic cumulative hazard function is used instead of H, we obtain a new distribution that is improper. In addition, to reduce the number of parameters, the substitution *υ *= *θ*^2-α ^is used [12]. The survival function of the new distribution is constructed as:

(3) $$\begin{array}{c} S\left(t;\lambda ,\tau ,\theta ,\alpha \right)=\\ \exp\left\{-\frac{\theta^{2}}{\alpha }\left[{\left(\frac{\log\left(1+\lambda t^{\tau }\right)}{\theta }+1\right)}^{\alpha }-1\right]\right\} \end{array}$$

where the parameter space is *θ *> 0, *λ *> 0, *τ *> 0, -∞ <*α *< ∞. The survival function must be between zero and one, as shown in the Appendix. If *α *< 0, the survival function is improper. This is an important characteristic of CIF modeling that differs from the two-parameter log-logistic distribution and other distributions.

### Hazard function

The hazard function can be directly obtained from equation (3), as:

(4) $$\begin{array}{c} h\left(t;\lambda ,\tau ,\theta ,\alpha \right)=\frac{-\frac{d}{dt}S\left(t\right)}{S\left(t\right)}=\\ \frac{\theta \tau \lambda t^{\tau -1}}{1+\lambda t^{\tau }}{\left[\frac{\log\left(1+\lambda t^{\tau }\right)}{\theta }+1\right]}^{\alpha -1} \end{array}$$

**Figure 1:**
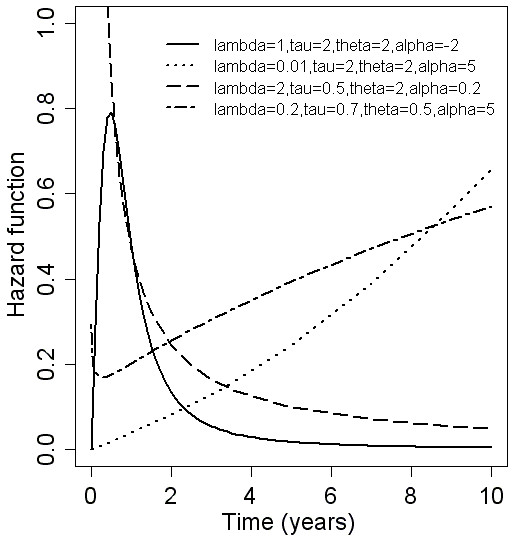
**Hazard function of the four-parameter log-logistic distribution**.

Because of the complexity of this hazard function formula, there is no simple mathematical expression for different types of hazard function. The flexibility of the hazard function is shown in Figure 1. Compared to the two-parameter model, the four-parameter log-logistic distribution has a flexible hazard function that can be monotonically decreasing or increasing, unimodal, or U-shaped.

### Cumulative incidence function

Competing risks data are represented as a pair (*T, δ*) where *δ *is the indicator variable, defined as *δ *= 0 if the observation is censored, and as *δ *= 1,2,...,*K *where *K *is the number of competing events. *T *is the time to first event or censoring. The two major quantities in the analysis of competing risks data are CSHF and CIF. The CSHF rate for event *k *is the instantaneous event rate for an individual who experiences event *k *at time *t *given that the subject experiences no other type of event up to *t*. The CIF for event *k*, *F_k_*(*t*) = *P*(*T *≤ *t*, *δ *= *k*), is the cumulative probability of observing event *k *by time *t*. The CIF for event *k *is defined as follows:

(5) $$F_{k}\left(t\right)=\int_{0}^{t}S\left(u\right)h_{k}\left(u\right)\;du$$

where *S(u) = P(T > u) *and *h_k_(u) *is the hazard function for the *k*th cause-specific event. In the literature, parametric methods are proposed to estimate CIF with the CSHF method [5,9,13]. Here we have also used the CSHF method to model CIF.

To estimate the CIF non-parametrically, the overall survival function should be replaced with the Kaplan-Meier estimate and the cause-specific cumulative hazard function with the Nelson-Aalen estimate [3].

### Estimation method

For convenience, we have assumed throughout this paper that there were two events: the desired event *k *= 1 and a competing event *k *= 2; and that *n *is the sample size. Because the two event are mutually exclusive, the overall survival function factored into a product of two cause-specific survival functions, i.e. *S(t, ψ) = S_1_(t,ψ_1_) S_2_(t, ψ_2_)*. Therefore, the likelihood function of the parametric inference is constructed as:

(6) $$\begin{array}{l} L(\psi_{1},\psi_{2})=\\ \prod_{i=1}^{n}(f_{1}{\left(t_{i},\psi_{1}\right)}^{\delta_{1i}}f_{2}{\left(t_{i},\psi_{2}\right)}^{\delta_{2i}}\\ \;S_{1}{\left(t_{i},\psi_{1}\right)}^{1-\delta_{1i}}S_{2}{\left(t_{i},\psi_{2}\right)}^{1-\delta_{2i}}) \end{array}$$

where *ψ_k _*= (*λ_k_*, *τ_k_*, *θ_k_*, *α_k_*) is the parameter vector for event *k*, *S_k_*(*t*, *ψ_k_*) is the survival function for event *k*, and *f_k_*(*t*, *ψ_k_*) is the density function of event *k *based on a four-parameter log-logistic distribution.

If event *k *occurs, *δ_ki _*= 1; otherwise *δ_ki _*= 0 (*k *= 1,2, *i *= 1,2,...,*n*). The covariance matrix, $I^{-1}\left({\hat{\psi }}_{1},{\hat{\psi }}_{2}\right)$, is estimated by the inverse of the Fisher information matrix [14]. According to the invariant property of the maximum likelihood estimate (MLE), the CIF is estimated by substituting $\hat{\psi }$ in expression (5), which yields ${\hat{F}}_{k}\left(t\right)=\int_{0}^{t}Ŝ\left(u\right){ĥ}_{k}\left(u\right)\;du$.

### Simulation study

A simulation study was used to compare the cumulative incidence estimate of the proposed distribution with a three-parameter distribution proposed by Sparling [8] and the non-parametric method at different times. As described by Beyersmann in 2009, we first simulated survival times *T *with all-cause hazards *h_1_(t) + h_2_(t) *on the basis of a two-parameter log-logistic distribution, with *λ_1 _*= 0.3, *τ*_*1 *_= 2.97 for the event of interest and *λ_2 _*= 0.03, *τ_2 _*= 1.1 for the competing event (based on fertility data). The event type was then determined by a binomial experiment with probability *h_1_(t)/(h_1_(t) + h_2_(t)) *on event type 1 [15,16]. Additionally, we generated censoring times with a binomial experiment. The data sets were simulated with sizes *n *= 1000, and a 7% censoring level. Using the data thus produced, we applied the four-parameter log-logistic, Sparling distributions, and non-parametric method to these data. Accordingly, 1000 samples were generated and the bias and empirical mean square error (MSE) of the CIF at time *t *were calculated as follows:

$$bias_{t}=\overset{1000}{\underset{j=1}{\sum }}\left({\hat{F}}_{1j}\left(t\right)∕1000\right)-F_{1}\left(t\right)$$

$$MSE_{t}=\sum_{j=1}^{1000}{\left(F_{1}\left(t\right)-{\hat{F}}_{1j}\left(t\right)\right)}^{2}/1000$$

where *F_1_(t) *is the true value of CIF at time *t *[17].

To test the efficiency of the parametric distribution proposed here, we used another simulation study. Failure times were generated on the basis of a two-parameter Weibull distribution with *k_1 _*= 1.4, *p*_*1 *_= 0.45 for the event of interest and *k_2 _*= 1.04, *p_2 _*= 0.03 for the competing event. We used the same method to fit the new distribution to these data.

The maximum likelihood estimates of the parameter vectors were calculated by PROC NLMIXED in SAS v. 9.1, and the non-parametric estimate of CIF was obtained with the "cuminc" R function from the "cmprsk" library. Because the determination of a suitable initial value to fit the models is an important problem in numerical studies, many initial values were examined to find a suitable convergence.

## Results

Table 1 summarizes the results of the first simulation in which the four-parameter log-logistic, Sparling distribution and non-parametric methods were fit for different times with *n *= 1000. The results showed that the bias and MSE of the CIF estimates obtained with the four-parameter method for the event of interest at *t *= 1.25 to *t *= 2 were smaller than with the Sparling distribution and the non-parametric method. For the competing event, the bias and MSE of the CIF estimates were lower than with the non-parametric method.

**Table 1:** The results of parametric and non-parametric estimates of CIF based on a four-parameter log-logistic and Sparling simulation for different times.

	**Time**						
	0.75	1.00	1.25	1.50	2.00	3.00	5.00
	
True value of CIF for event 1	0.11	0.23	0.36	0.49	0.68	0.85	0.92
	
Distribution							
Four-parameter log-logistic							
CIF	0.06	0.18	0.32	0.45	0.64	0.82	0.91
Bias	-0.05	-0.05	-0.04	-0.04	-0.04	-0.03	-0.01
MSE × 10^2^	0.30	0.30	0.20	0.20	0.20	0.10	0.01
Sparling							
CIF	0.07	0.17	0.30	0.44	0.65	0.83	0.91
Bias	-0.04	-0.06	-0.06	-0.05	-0.03	-0.02	-0.01
MSE × 10^2^	0.17	0.40	0.39	0.32	0.12	0.05	0.02
Nonparametric							
CIF	0.07	0.18	0.31	0.44	0.64	0.82	0.91
Bias	-0.04	-0.05	-0.05	-0.05	-0.04	-0.03	-0.01
MSE x10^2^	0.20	0.27	0.26	0.29	0.22	0.10	0.02
	
True value of CIF for event 2	0.020	0.030	0.033	0.037	0.043	0.050	0.052
	
Distribution							
Four-parameter log-logistic							
CIF	0.052	0.054	0.055	0.055	0.056	0.057	0.057
Bias	0.032	0.024	0.022	0.018	0.013	0.007	0.005
MSE × 10^2^	0.100	0.100	0.010	0.040	0.020	0.010	0.010
Sparling							
CIF	0.048	0.053	0.056	0.058	0.060	0.061	0.062
Bias	0.028	0.023	0.023	0.021	0.017	0.011	0.010
MSE × 10^2^	0.100	0.100	0.100	0.100	0.040	0.020	0.020
Nonparametric							
CIF	0.059	0.059	0.059	0.059	0.059	0.059	0.059
Bias	0.039	0.029	0.026	0.023	0.016	0.009	0.007
MSE × 10^2^	0.150	0.100	0.070	0.050	0.030	0.010	0.010

The true model is a two-parameter log-logistic distribution.

The results of the second simulation are summarized in Table 2. Up to *t *= 1.5, the bias and the MSE of the CIF estimates obtained with the non-parametric method for the event of interest were lower than with the four-parameter method, but after *t *= 2, the bias and MSE of the CIF estimates for the competing event with the new distribution were equivalent or slightly lower than with the non-parametric method. For the competing event, the bias and MSE of the CIF estimates were lower than with the non-parametric method at all times.

**Table 2:** The results of parametric and non-parametric estimates of CIF based on a four-parameter log-logistic simulation for different times.

	**Time**						
	0.75	1.00	1.25	1.50	2.00	3.00	5.00
	
True value of CIF for event 1	0.19	0.27	0.35	0.43	0.56	0.75	0.91
	
Distribution							
Four-parameter log-logistic							
CIF	0.13	0.21	0.29	0.37	0.52	0.73	0.89
Bias	-0.06	-0.06	-0.06	-0.06	-0.04	-0.02	-0.02
MSE × 10^2^	0.42	0.49	0.47	0.45	0.22	0.06	0.04
Nonparametric							
CIF	0.14	0.22	0.30	0.38	0.52	0.72	0.89
Bias	-0.05	-0.05	-0.05	-0.05	-0.04	-0.03	-0.02
MSE × 10^2^	0.26	0.25	0.26	0.29	0.23	0.14	0.05
	
True value of CIF for event 2	0.017	0.023	0.027	0.031	0.037	0.046	0.051
	
Distribution							
Four-parameter log-logistic							
CIF	0.021	0.027	0.032	0.036	0.043	0.052	0.058
Bias	0.004	0.004	0.005	0.005	0.006	0.006	0.007
MSE × 10^2^	0.003	0.003	0.010	0.010	0.010	0.010	0.010
Nonparametric							
CIF	0.014	0.014	0.036	0.036	0.049	0.055	0.058
Bias	-0.003	-0.009	0.009	0.005	0.012	0.009	0.007
MSE × 10^2^	0.002	0.010	0.010	0.010	0.020	0.010	0.010

The true model is a two-parameter Weibull distribution.

In summary, these two simulations indicate that the four-parameter modeling of CIF was as efficient as the non-parametric method and the Sparling distribution and sometimes led to better estimates of CIF. Moreover, the four-parameter log-logistic model performed well under a Weibull distribution.

### Example: women's fertility history

We tested the proposed distribution on a set of real data. In a cross-sectional study, the fertility history of 858 women aged 15-49 years in rural areas of the Shiraz district (southwestern Iran) was reviewed (unpublished data). The women were selected by multistage random sampling from a list of villages in 2008. Only the first pregnancy of each woman was included in this study. A self-administered questionnaire regarding fertility history was used. After women with an undesired first pregnancy were excluded, the final sample consisted of 652 women. Live birth as a result of the first delivery was our desired event, and a stillborn fetus or abortion was the competing event. The event time was defined as the interval between marriage and a live birth, a competing event or censoring. Also, women who had not given birth on the date of interview (7% in this data set) were censored.

**Figure 2:**
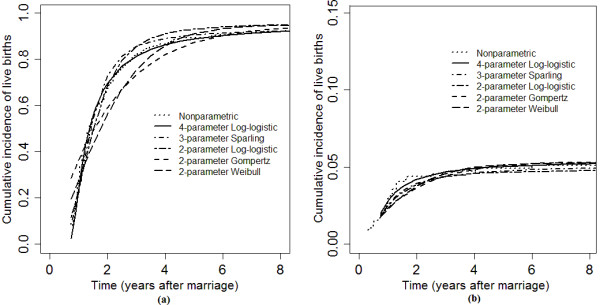
**Cumulative incidence function estimates of live births (a) and abortions (b) with the non-parametric and two- and four-parameter log-logistic, Weibull, Gompertz and Sparling distributions in a fertility history study**.

The estimated cumulative incidence of live births and abortions or stillborn fetuses based on the two- and four-parameter log-logistic, Weibull, Gompertz and Sparling distributions and the non-parametric estimates are shown in Figure 2. Up to time *t *= 3, the cumulative incidence of live births increased rapidly; thereafter, cumulative incidence tended to plateau. This means that the probability of live births during the first four years after marriage increased rapidly, and remained approximately constant thereafter. The curves also show that the four-parameter log-logistic distribution was closer to the non-parametric estimate than the other distributions at all times. For shorter intervals since marriage, the two-parameter log-logistic and Sparling distributions were closer to the non-parametric estimates than to the Weibull and Gompertz distributions. After *t *= 5, all distributions were close to the observed data.

Table 3 shows the Akaike information criterion (AIC), Bayesian information criterion (BIC) and estimated cumulative incidence for two events in different times. Based on AIC and BIC criteria, the four-parameter log-logistic model with the lowest AIC and BIC showed a better fit to the data than the two-parameter log-logistic, Sparling, Weibull or Gompertz distributions. Because the two-parameter log-logistic distribution is nested within the Sparling and the four-parameter log-logistic distributions, we can compute likelihood-ratio chi-square statistics to test the fit of the nested models. The likelihood-ratio chi-square statistics and their corresponding p-values are:

**Table 3:** The Akaike information criterion (AIC), Bayesian information criterion (BIC) and the estimates of the cumulative incidence function under competing risks based on different distributions with the non-parametric method.

	**Time (years)**								
**Distribution**	**0.75**	**1**	**1.5**	**2**	**3**	**5**	**10**	**AIC**	**BIC**
	
Two-parameter log-logistic								1894.0	1912.0
Live birth	0.1145	0.2317	0.4946	0.6857	0.8556	0.9307	0.9497		
Stillborn fetus or abortion	0.0189	0.0246	0.0333	0.0375	0.0457	0.0514	0.0477		
Four-parameter log-logistic								1685.3	1721.1
Live birth	0.0257	0.2373	0.5552	0.6949	0.8133	0.8876	0.9274		
Stillborn fetus or abortion	0.0200	0.0278	0.0370	0.0419	0.0467	0.0503	0.0525		
Two -parameter Weibull								2195.0	2212.0
Live birth	0.1942	0.2749	0.4292	0.5626	0.7532	0.9098	0.9472		
Stillborn fetus or abortion	0.0173	0.0225	0.0310	0.0372	0.0457	0.0507	0.0526		
Two -parameter Gompertz								2299.9	2317.9
Live birth	0.2862	0.3617	0.4890	0.5897	0.7317	0.8718	0.9425		
Stillborn fetus or abortion	0.0185	0.0231	0.0307	0.0365	0.0441	0.0507	0.0533		
three-parameter Sparling								1817.2	1856.0
Live birth	0.0856	0.2198	0.5416	0.7290	0.8539	0.9047	0.9242		
Stillborn fetus or abortion	0.0188	0.253	0.0345	0.0394	0.0439	0.0473	0.0499		
Nonparametric									
Live birth	0.0062	0.2601	0.5542	0.6723	0.8194	0.8934	0.9287		
Stillborn fetus or abortion	0.0170	0.0279	0.0405	0.0437	0.0455	0.0490	0.0535		

χ^2 ^= 69.2, df = 1, p < 0.001 for two-parameter log-logistic versus Sparling and χ^2 ^= 217.1, df = 2, p < 0.001 for two-parameter log-logistic versus four-parameter log-logistic. Likelihood-ratio test, AIC and BIC show the four-parameter log-logistic distribution fits the data better than two-parameter log-logistic and Sparling distributions. These results confirm the findings in Figure 2, and again indicate that the proposed distribution shows a closer fit to the observed data than the other distributions to which it is compared.

## Discussion

Although non-parametric methods such as the Kaplan-Meier approach are widely used in survival analysis and may show a very close fit to the data, they do not provide additional information about the nature of the data. Therefore, in this study our ultimate aim was to develop a new parametric distribution by extension of the two-parameter log-logistic distribution. The addition of third and fourth parameters allows the model to capture U-shaped hazards.

Our simulation study showed that the parametric estimate of CIF with the new distribution was slightly less biased and had a smaller MSE than the estimate obtained using non-parametric methods. Simulations with the two-parameter log-logistic and Weibull distributions showed that our proposed four-parameter distribution had appropriate efficiency. Also, analyses of real data indicated that the proposed distribution showed a much better fit to the data than the other distributions tested. Our results are consistent with other studies in finding that an appropriate parametric model yields more precise estimates of cumulative incidence than non-parametric methods, and is thus a potentially suitable way to describe quantities of competing risks [9,18]. In contrast, if a parametric model is mis-specified, the quantities will be estimated incorrectly, which will clearly bias the inferences [12]. However, our proposed distribution captures various hazard shapes well, which extends its applicability to a variety of survival data.

In addition to this advantage, the proposed distribution is improper for *α *< 0. This property makes our proposed distribution superior to other distributions such as the Weibull, two-parameter log-logistic, three-parameter Sparling and generalized Weibull models [6,8]. This characteristic of our distribution also makes it possible to evaluate the direct effect of covariates on CIF, which is not possible in the CSHF model [19,20]. The potential applications of direct modeling of CIF and parametric regression models with the four-parameter log-logistic distribution will be examined in forthcoming papers.

## Conclusions

Despite the complexity of this distribution for modeling CIF (which is one of its limitations), the results of our simulation study and real-data application show that the new distribution achieves a much better fit to the data than other distributions that use fewer parameters. Whereas the two-parameter log-logistic is a proper distribution, the four-parameter log-logistic is an improper distribution in the subset of parameter space. Therefore, this distribution is suitable for parameterizing CIF directly in competing risk models. Moreover, it is can be added to a family of distributions and also potentially useful for parameterizing survival data in general.

## Appendix

The survival function of the new distribution is as follows:

$$\begin{array}{c} S\left(t;\lambda ,\tau ,\theta ,\alpha \right)=\\ \exp\left\{-\frac{\theta^{2}}{\alpha }\left[{\left(\frac{\log\left(1+\lambda t^{\tau }\right)}{\theta }+1\right)}^{\alpha }-1\right]\right\} \end{array}$$

The parameter space is θ > 0, τ > 0, λ > 0, -∞ < α <∞. The survival function must be between zero and one for all values in the parameter space. If (*θ*^2^[(log(1+*λt^τ^*)/*θ*+1]*^α^*/*α*-1) > 0, then the condition holds. First, if α > 0, log(1+*λt^τ^*)/*θ *+ 1 must be positive, which implies that log(1+*λt^τ^*)/*θ *> 0 since λ > 0, τ > 0 and θ > 0, log(1+*λt^τ^*)/*θ *is always positive. Thus, the condition holds for α > 0. The same result follows for the case α < 0.

## List of abbreviations

CIF: cumulative incidence function; CSHF: cause-specific hazard function MSE: mean square error; MLE: maximum likelihood estimate; AIC: Akaike information criterion; BIC: Bayesian information criterion.

## Competing interests

The authors declare that they have no competing interests.

## Authors' contributions

ZS and NZ were responsible for the design, simulation, analysis and interpretation. SMTA supervised the study and interpreted the results. All authors read and approved the final manuscript.

## Authors' information

Corresponding author: SMT Ayatollahi, Ph.D., FSS, C.Stat. Professor of Biostatistics, The Medical School, Shiraz University of Medical Sciences, Shiraz, Islamic Republic of Iran. P.O.Box 71345-1874.
